# Disease associations for rare immune genetic variants require statistical or rule-based support

**DOI:** 10.70962/jhi.20260099

**Published:** 2026-10-19

**Authors:** Paul E. Gray, Stuart G. Tangye, Edwin P. Kirk

**Affiliations:** 1 School of Medicine, Western Sydney University, Sydney, Australia; 2 Department of Immunology and Infectious Diseases, Sydney Childrenʼs Hospital, Sydney, Australia; 3 Garvan Institute of Medical Research, Sydney, Australia; 4 Faculty of Medicine and Health, School of Clinical Medicine, UNSW Sydney, Sydney, Australia; 5 Centre for Clinical Genetics, Sydney Childrenʼs Hospital, Sydney, Australia; 6 NSW Health Pathology Randwick Genomics Laboratory, Australia Garvan Institute of Medical Research, Sydney, Australia

## Abstract

Validating weak gene–disease associations requires statistical support from large case–control studies. In contrast, strong (e.g., monogenic) gene–disease associations may be established from relatively few affected individuals, when independent orthogonal evidence converges. Here, we examine four publications associating heterozygous variants in five genes—*TNIP1*, *P2RY8*, *BLK*, *BANK1*, and *SH2B3*—with autoimmune disease (predominantly systemic lupus erythematosus [SLE]). While we do not dispute the molecular findings of these studies, we do not accept the proposed disease associations, which lack case–control evidence and where high minor allele frequencies in non-disease populations, indicate that the genotypes are far too common to represent Mendelian disease alleles. Additionally, 8 of 18 alleles were searchable in UK Biobank, and none showed enrichment in SLE. Until further evidence emerges, caution is warranted in ascribing disease association to heterozygous genotypes in *TNIP1*, *P2RY8*, *BLK*, *BANK1*, and *SH2B3*, and linkage to patients in clinical pedigrees should not be taken as inferring a monoallelic inborn error of immunity.

## Teaser

Validating weak gene–disease associations requires statistical support from large case–control studies, whereas powerful associations may be demonstrated by adhering to well-established rules for confirming a monogenic association. This article analyses claims of gene–disease association for *TNIP1*, *P2RY8*, *BLK*, *BANK1*, and *SH2B3*, where these rules have not been applied, and where the association is therefore not supported.

## Introduction

The relationship between genetic variants and human phenotypes is complex and spans a spectrum from highly penetrant Mendelian disorders at one extreme, through to quantitative trait loci (QTL) with very small individual impacts on phenotype at the other. This is by no means a binary distinction; there can be considerable heterogeneity in effect between different variants, even in the same gene. Individual genetic variants may have relatively strong effect, to the extent of being arguably Mendelian, but with incomplete penetrance (and often associated with an attenuated phenotype in those individuals who are affected). Conversely, they may be clearly disease associated, but only as part of oligogenic causation, or in the setting of specific environmental exposures.

Methodologies used to define gene–disease associations differ depending on the size of the effect the genetic variant has on the disease phenotype and the patient population under study. On one hand, weak associations—where the variant contributes only a small percentage of the genetic predisposition—require the statistical power of large association studies (e.g., thousands of individuals) to confirm the effect. There is published guidance on sample sizes needed to detect effects of varying strength given the population frequency of the variant(s) under study (1). On the other hand, population or case/control studies are usually not needed to define powerful gene–disease associations in which a single genetic variant is responsible for most, if not all, of the disease predisposition (i.e., monogenic disease). By building an orthogonal case using independent but interconnecting pieces of evidence, a gene–disease association for monogenic or highly penetrant genotypes can be validated in the absence of the statistical power derived from larger cohorts. For inborn errors of immunity (IEIs), the standards for attributing pathogenicity to novel genes identified in small numbers of individuals were outlined in a seminal article in 2014 by Casanova and colleagues (2). The guidelines include several stipulations of relevance here:

1. The candidate genotype should appear to be monogenic.
2. Population studies should indicate that the candidate genotype does not occur in healthy individuals.
3. The frequency of the candidate genotype/variant should not exceed the frequency of the clinical phenotype (i.e., disease incidence).
4. If the candidate genotype is loss of function (LOF), the frequency of other variants resulting in LOF (e.g., nonsense/frameshift) in the general population must not exceed the frequency of the clinical phenotype.
5. The causal relationship between the candidate genotype and the clinical phenotype should be established via cellular or animal models (2).

Of course, in many instances it will not be possible to generate a sufficiently large cohort to provide the statistical power to demonstrate the enrichment of disease-associated non-monogenic variants/genotypes, potentially because the variant is rare, the effect size is small, or the patient population is infrequent. In such instances, the association may remain unproven.

In vitro functional evidence and animal models may contribute to establishing a causal role for variants in a given gene. However, if a variant is either too common to be plausibly disease-associated, or is present at the same frequency in cases and in controls, the population data necessarily carry more weight than other types of evidence. If we put aside the importance of functional or animal model data in the papers discussed below, this is not to imply that the experiments have not been conducted correctly. However, it is possible that observations made in vitro do not reflect the reality of biology in vivo. Alternately, there may be a real functional impact, but this may not lead to disease, for example, because the biological pathway has sufficient redundancy that alteration to the function of one protein is fully compensated by the function of others. Similarly, observations in nonhuman species are not always reflective of human physiology or pathophysiology. If these considerations are not kept in mind when evaluating a possible new gene–disease association, there is a risk of either erroneously assigning a causative role to variants that do not actually cause disease; or of over-estimating the strength of the effect of variants.

Here, we discuss recent publications that we assess as representing examples of this problem. In each report, heterozygous variants in genes not previously linked to monoallelic diseases, are identified as having a causative role for autoimmune disease in studies of small numbers of patients. The strength of the association is not always stated by the authors as being Mendelian, but the evidence is presented in a way that at least implies a strong effect. However, for each of *TNIP1* (3), *P2RY8* (4), *BLK*, *BANK1* (5), and *SH2B3* (6), the available population data either make a disease association unlikely or indicate that any effect is much weaker than implied or stated by the authors. In none of these cases was an adequate statistical case made for a gene–disease association, and the authors relied on constructing an orthogonal case, including in some papers by presenting apparent segregation in family pedigrees, as if they were variants with powerful effect. However, the minor allele frequency (MAF) of most of these candidate variants in population databases is far too high to support any role for the variant in driving individual clinical phenotypes without the use of an appropriately powered association study. Critically, while almost half of the variants in these studies (8 of a total of 18) were searchable in the UK Biobank (UKBB) (7), none demonstrated an association with systemic lupus erythematosus (SLE).

## Results

### TNIP1

The first study proposed a heterozygous variant in *TNIP1* (p.Q333P - rs148243052), identified in two unrelated individuals, as a driver of autoimmune disease (3). The authors do not explicitly claim a monogenic IEI, but they present pedigrees and claim a causative role for the variant, at least implying a strong effect. Not surprisingly, with only two cases there were no case–control statistics to support this claim. Thus, any disease association would have to rely on establishing an orthogonal case for TNIP1 p.Q333P as a powerful or Mendelian allele. Such an approach was highly unlikely to be valid, as the clinical phenotype of the patients was not suggestive of monogenic disease. For instance, a 47-year-old Chinese female with SLE (3) is well outside the spectrum of presentations considered likely to represent monogenic lupus (8). More importantly, while the paper by Medhavy et al. described TNIP1^Q333P^ as ‘ʻultrarare”(3), it actually constitutes 36 of 44,892 alleles from East Asia in the gnomAD database v4.1.1 (9). In other words, ∼1 in 620 East Asians (EASs) are heterozygous carriers of this proposed pathogenic allele. Carrier frequencies are similar in EASs in the All of Us (1/540, USA) and jMorp (1/810, Japanese) (10) population databases. Based on these frequencies, if the heterozygous *TNIP1*^Q333P^ variant caused a highly penetrant Mendelian autoimmune disease by haploinsufficiency, disease prevalence would need to be ∼1/500–1,000 individuals of the EAS origin, and TNIP1^Q333P^ would have to be responsible for most cases. Even if the allele demonstrated incomplete penetrance, this would still be one of the most common Mendelian conditions in East Asia, and indeed the world, which is clearly not the case. In fact, this variant is too common in population databases to claim even a weak disease association without large-scale case–control statistics. In this context, it is difficult to interpret the mouse data presented by the authors, and while it remains possible that TNIP1^Q333P^ may be relevant to human disease, at present any such link is speculative. If there is an effect, it could only be very weak, with the variant making a minor contribution to disease causation.

### P2RY8

The second paper began with a cohort of 61 Chinese trios in which the probands had childhood-onset SLE (4). Whole-exome sequencing identified a novel de novo heterozygous missense variant in *P2RY8* (p.Leu257Phe; L257F) in an affected female (4). *P2RY8* is located in the pseudo-autosomal region of the X and Y chromosomes and was not previously associated with SLE. The heterozygous P2RY8^L257F^ variant is genuinely rare and the authors present evidence that it causes loss of P2RY8 function, even referring to this as haploinsufficiency. However, there is compelling preexisting evidence that heterozygous LOF variants in *P2RY8* do not predispose to SLE. Firstly, the gene has a low probability of LOF intolerance score in gnomAD (0.04), which augurs against a haploinsufficient effect. Of course, this itself does not exclude a role for heterozygous LOF variants in disease pathogenesis, given the gene is relatively small and the expected number of variants is also small, and predisposition to SLE may not produce a strong enough selective pressure to remove LOF variants from the population. The key evidence against *P2RY8* haploinsufficiency as a mechanism for disease pathogenesis comes from Turner syndrome (45,X). Although autoimmune disease in general is much more common in women with Turner syndrome than in women with two X chromosomes (11), the reverse is true for SLE (12). Since women with Turner syndrome have only a single copy of the X chromosome, and hence this gene, there is already compelling evidence that heterozygous loss of P2RY8 function does not predispose to SLE. Thus, in relation to the paperʼs reference to haploinsufficiency, we can say with near certainty that this is not a haploinsufficient gene with respect to SLE causation.

Following the initial finding of the P2RY8^L257F^ variant, the team of He et al. searched for additional variants in *P2RY8* in other autoimmune cohorts to support their case for pathogenicity (4). Analysis of whole-exome data from 2,000 Australian subjects enriched for autoimmunity identified one affected individual with a P2RY8 N97K variant inherited from a clinically well mother. Additionally, data from SLE cohorts from China, Australia, and the United States identified six individuals—all of Chinese ancestry—heterozygous for the EAS restricted P2RY8 E323G variant, which was shown to be enriched in EAS SLE cases vs. the variant minor allele frequency (MAF) in gnomAD, at the 5% level of significance. However, while this appears to represent a statistical disease association, this should be treated with caution. The authors state that this was the only rare variant (RV) identified in the cohort of 725 individuals with SLE. However, P2RY8^E323G^ is not particularly rare—it is present in 1 in 480 EAS individuals in gnomAD v4.1.1 and 1 in 340 in the All of Us database. Additionally, there are 16 other missense variants on gnomAD that would fulfil the authorsʼ definition of rare (MAF < 0.005), that are more common than P2RY8^E323G^, and could have been assessed. Without the knowledge of which other variants that may have been considered but not selected for further study, it is difficult to know for certain whether a statistical adjustment for multiple comparisons should have been applied, but this seems likely. Any such adjustment would have rendered the apparent association (with P = 0.0423) nonsignificant. Even without that information, in the absence of a replication cohort, it is difficult to place any weight on the apparent association.

Thus, in the absence of a valid statistical association, and evidence from Turner syndrome that haploinsufficiency of P2YR8 is not associated with SLE, the functional evidence showing that the N97K and E323G variants cause incomplete reduction in P2RY8 activity is uninformative in relation to a possible role for these variants in disease predisposition.

Despite the lack of evidence for a gene–disease association, the study by He et al. implicating *P2RY8* variants in SLE (4) has already caused confusion in the literature. The common P2RY8^E323G^ variant was identified by a separate group in two related Vietnamese patients with cutaneous lupus (13). This group interpreted the variant as a strong but not monogenic susceptibility allele in a report entitled “Navigating the boundary between monogenic disease and genetic susceptibility to lupus,” which relied heavily on the association between *P2RY8* variants and SLE reported by He et al. (4). We would argue there is currently insufficient evidence for any association between variants in *P2RY8* and SLE, even at the level of a low penetrance susceptibility allele.

### *BLK *and *BANK1*

This study was different from the *TNIP1* and *P2RY8* studies, in that it began with a case/control analysis, an RV burden analysis on a panel of 76 SLE-related genes, which showed a modest apparent difference in RV frequency between 133 SLE patients (82%) and 97 healthy controls (HC) (72%) (5). *BLK* was chosen for further analysis based on a higher frequency of RV in SLE (10.5%) vs. HC (7.2%) (5), although the authors themselves note that there is no difference in the frequency of RV in BLK between cases and controls (P = 0.5) (5). Two variants in a second gene, *BANK1*, were additionally chosen for study without a statistical basis, but rather because it was proposed these variants might contribute to the disease phenotype, either alone or in epistasis with a *BLK* variant (5). This latter concept derived from a 2012 study that provided evidence for an epistatic relationship of variants in *BLK* and *BANK1* in SLE (14).

With no statistical evidence to support the concept that “variants in BLK and BANK1 contribute to human lupus,” Jiang et al. proceeded to build a case for a disease association by linking individual heterozygous *BLK* and *BANK1* genotypes to individual members of family pedigrees. They stated that “BLK and BANK1 variants segregate with autoimmune disease,” implying a powerful causative effect, while acknowledging segregation was incomplete in some families, describing the variants as hypomorphic.

However, the identification of variants across multiple individuals in multiple families by Jiang et al. is far less likely to reflect segregation with disease than the frequency of the alleles under study. The heterozygous *BLK* variants highlighted in Jiang et al. (5) are predicted to have a combined global prevalence of ∼300 million individuals, while ∼260 million people worldwide are predicted to be heterozygous for the BANK1^W40C^ allele alone (Table 1). By comparison, the estimated global prevalence of SLE is ∼3.4 million cases (43.7/100,000) (15). This suggests a maximum disease penetrance of <1% for *BLK* or *BANK1* variants, assuming they are associated with all cases of SLE, and that Jiang et al. identified all putative disease-associated variants in a modestly sized cohort of 133 patients. Clearly, to robustly validate such a weak disease association, a vastly larger cohort of patients would be needed. Additionally, four of the variants claimed as segregating with disease in a heterozygous state in individual family pedigrees—BLK^A71T^, BLK^R238Q^, BLK^R359C^, BANK1^W40C^—are present in the homozygous state in gnomAD (16), with a predicted global homozygous prevalence for BLK^A71T^ of circa (c.) 1 million and BANK1^W40C^ of c.2 million people (Table 1). Finally, we could query six of the nine variants in *BLK* and *BANK1* in UKBB, comparing allele frequencies for individuals with and without SLE. None demonstrated a disease association (7).

**Table 1.** Characteristics of *TNIP1*, *P2RY8*, *SH2B3*, *BLK*, and *BANK1 *variants

​	Variant	Number of alleles in major ethnicity on gnomAD 4.1.1	Het carrier frequency (major ethnicity) gnomAD	Number of homozygous carriers in gnomAD	Estimated total het carrier frequency	Estimated total homozygous carriers	P-value for association to SLE in UKBB
*TNIP1*	Q333P rs148243052	36/44,892 EAS	1:600 EAS	0	c.2.5 million EAS	c.1000 EAS	N/A
*SH2B3*	C133Y rs752374176	25/1,106,898 European	1:22,000 European	0	c.50,000 European	0	N/A
	E400K rs72650673	1,998/1,096,136 European	1:300 European	3	c.3.5 million European	c.3,000 European	N/A
	E208Q rs202080221	981/1,478,578 Global	1:800 Global	3	c.10 million Global	c.4,000 Global	0.14
	Q540X rs1874236975	2/1,614,172 Global	1:400,000 Global	0	c.20,000 Global	0	N/A
	R566Q rs148791142	259/1,179,770 European	1:2,200 European	0	c.500,000 European	c.50 European	0.64
*BLK*	A71T rs55758736	18,440/1,613,932 Global	1:40	156	c.200 million Global	c.1 million Global	0.88
	R131W rs73663163	1,279/1,179,984 European	1:500	0	c.2 million European	c.1,000 European	0.16
	R131Q rs144615291	59/1,180,028 European	1:10,000	0	c.100,000 European	c.2 European	N/A
	R238Q rs14185425	7,364/1,614,156 Global	1:100	24	c.80 million Global	c.200,000 Global	0.29
	R359C rs146505280	1,600/1,614,066 Global	1:500	3	c.16 million Global	c.8,000 Global	0.37
	P307R	0	0	0	0	0	N/A
	Y350H rs758750492	1/1,613,860 Global	1:800,000	0	c.10,000 Global	0	N/A
*BANK1*	D400G rs201960198	266/1,150,666 European	1:2,000	0	c.500,000 European	c.50	0.66
	W40C rs35978636	26,609/1,596,420 Global	1:30 Global	276	c.260 million Global	c.2 million Global	0.89
*P2RY8*	L257F	0	0	0	0	0	N/A
	N97K rs766166608	18/1,179,866 European	1:30,000 European	0	c.30,000 European	c.1 individual	N/A
	E323G rs777567195	47/44,874 EAS	1:500	0	c.3 million EAS	c.1,600 EAS	N/A

The columns list numbers of heterozygous (het) and homozygous individuals on gnomAD, predicted numbers of heterozygous and homozygous carriers in the population, and P values for variant association to SLE in the UKBB. Allele counts and carrier frequency from gnomAD 4.1.1 (16). Total global population (∼8 billion), and estimate of global peoples of European (∼1 billion) and EAS (∼1.66 billion) ethnicity, based on United Nations statistics (17). UKBB associations are imputed using TOPMed with comparisons derived from the SAIGE modeling tool (18).

Regarding the possibility of epistasis between variants in *BLK* and *BANK1* facilitating disease in a more penetrant manner, the original study from which this idea arose (14) was very different from the paper of Jiang et al., in that it provided a statistical case for an epistatic relationship between *BLK* and *BANK1* variants in SLE based on a meta-analysis of 2,318 European SLE cases with a similar sized control group (14). Castillejo-Lopez et al. did not analyze any of the *BLK* or *BANK1* variants studied by Jiang et al. and, importantly, did not imply a powerful variant–disease association of common variants by showing them segregating in individual patients in family pedigrees (14).

### SH2B3

The final study was related to the study by Jiang et al. with the choice of genes deriving from the same RV burden analysis on a panel of 76 SLE-related genes from the same SLE cohort. Zhang et al. chose heterozygous *SH2B3* variants for further analysis because they were present in 5.26% of SLE vs. 2.06% of HC, which again does not reach statistical significance (Chi square P = 0.2) (6). Once again, in the absence of a statistical basis for an association, the authors linked variants to family pedigrees and referred to the variants as hypomorphic, although in this case the authors did not explicitly state that the variants segregated with disease and suggested the presence of the variant alone was insufficient, with additional environmental or genetic triggers being needed to initiate disease.

However, the concept that these “variants in lupus patients predispose to autoimmunity” is not realistic (6), given the lack of statistical support and the fact that the three most common heterozygous *SH2B3* variants highlighted by Zhang et al. are predicted to be carried by almost 32 million individuals (Table 1), 10 times more than the estimated global prevalence of SLE itself (15). In this context, demonstrating an association would again require a large case/control study, especially since the two variants that were searchable in UKBB also showed no association with SLE (Table 1).

Additionally, there is the significant potential for over-interpreting clinical possibilities when linking these heterozygous *SH2B3* variants to disease in family pedigrees, because biallelic *SH2B3* LOF variants cause a severe autosomal recessive IEI manifesting as hematological malignancy and immunodeficiency (19, 20). Two of the variants examined by Zhang et al.—*SH2B3*^E400K^ and *SH2B3*^E208Q^ (16)—are present in the homozygous state in gnomAD, with thousands of people predicted globally to be homozygous (Table 1). While Zhang et al. argue that these alleles are hypomorphic, the inclusion of family pedigrees implies there is a genotype–phenotype relationship at an individual level and raises concerns that individuals identified as carrying one or more of these variants, with or without a true LOF variant in *SH2B3*, may be incorrectly diagnosed with a rare and severe immune disease (19).

## Discussion

While the range of effect sizes of genetic variation on disease pathogenesis is extremely broad, when it comes to demonstrating a gene–disease or variant–disease association, despite myriad technologies, the available strategies remain limited. The researcher can either conduct an association or targeted case–control study that demonstrates statistical enrichment of the gene/variant with disease, or for monogenic variants, associations can be demonstrated by strict adherence to well-established rules for validation.

For genes and inheritance patterns that are already disease-associated, where we are querying a specific variant, we can apply clinical criteria (e.g., American College of Medical Genetics and Genomics [ACMG] [21]), which include a stipulation around rarity. An example of a published variant failing clinical validation is the p.T647P variant in *TNFAIP3*, which has been associated with an autoimmune predisposition known as haploinsufficiency of A20 (HA20). TNFAIP3^p.T647P^ was reported as causing a monogenic predisposition to autoimmunity in three members across three generations of one family (22). While the clinical phenotype was consistent with HA20, and the variant segregated with autoimmune disease in the kindred (22), the p.P647T variant is carried by 1/500 people in gnomAD v4.1.1, with eight homozygotes in the All of Us database. These population data trump the other findings in that paper (22). With the population data and the variant’s low in silico scores (AlphaMissense 0.06, REVEL 0.03), it is classified as Likely Benign using the current ACMG framework for variant classification (BS1, BP4_moderate).

By contrast, when we are trying to identify a previously undescribed monogenic gene–disease association, there are established criteria that must be applied (2). One of the primary rules is that if the candidate genotype is to be considered as a monogenic causation of disease, then the candidate variant, or genotypes with the same effect (e.g., complete LOF), should not be present at appreciable frequency in healthy individuals (2), a stipulation which has been made easier to assess by databases of genetic variation (e.g., gnomAD [9], jMorp [10]). There are of course exceptions, in which it has been possible to associate a more common variant to a rare disease, with a classic example being the autosomal recessive condition thrombocytopenia absent radius (TAR) syndrome. In TAR, common hypomorphic single-nucleotide polymorphisms (SNPs) in *RBM8A* which have a combined population frequency of 3.4% are necessary to bring about clinical disease, but only when *in trans* with a rare deletion (23). Similarly, in familial Mediterranean fever (FMF) the interaction of a common low-impact SNP in *MEFV* (e.g., E148Q) with rarer high-impact variants (e.g., M694V) *in trans* or even potentially *in cis*, may result in a disease phenotype (24). Of course in the case of *RBM8A*, the genotype under study is the combination of the rare and common variants, which together are rare and are not more common than the clinical disease (23), while FMF is a relatively common genetic disease in which genotype comparison of large numbers of cases and controls has been possible, although even then the impact of the E148Q variant remains controversial (25).

In each of the examples of *TNIP1*, *P2RY8*, *BLK*, *BANK1*, and *SH2B3* discussed above (3, 4, 5, 6), there is neither sufficient evidence for highly penetrant Mendelian disease causation nor statistical support for a variant effect within the spectrum that ranges from QTL, through risk allele to hypomorphic variant. In each instance, there is evidence against a role for the reported disease associations, either through population frequency, case–control evidence accessible via the UKBB data, or in one case through a very well-studied natural experiment, Turner syndrome. The carefully conducted functional and animal studies presented in these studies (3, 4, 5, 6) raise questions about possible biological effects of the variants, and the possibility that some variants in these genes may contribute to disease has not been excluded. However, until new evidence emerges, no causal role should be attributed to any of the heterozygous variants in *TNIP1*, *P2RY8*, *BLK*, *BANK1*, or *SH2B3*, discussed in these papers, or indeed to other variants in these genes—except in relation to previously established gene–disease relationships with other mechanisms. Importantly, identification of such variants should not be used to guide management decisions for individual patients.

### Conclusion

There are profound risks to patients from incorrectly assigning a causal role to the incorrect genetic variant. These can include inappropriate therapeutic decision making, failure to correctly diagnose an alternative cause for the patient’s phenotype, and inappropriate advice regarding implications for family members. While there are many new technologies for querying variant–disease associations, proving such associations will always rely on either identifying a statistically significant difference between cases and controls, or adhering to strict guidelines for confirming a powerful genotype–phenotype relationship in the absence of population statistics. Thus, it will remain difficult to link rare non-monogenic variants to disease, because progressing an orthogonal case for a gene–disease association is not a valid approach if the variant is present in appreciable numbers of controls, and especially if the candidate genotype is more common than the disease under study. The studies of heterozygous variants in *TNIP1*, *P2RY8*, *BLK*, *BANK1*, and *SH2B3* discussed here (3, 4, 5, 6), do not provide either a convincing statistical or orthogonal case and these putative gene–disease associations should be considered unproven. They should not be used in patient management.

## Materials and methods

The arguments presented here rely on open-source data from databases including gnomAD 4.1.2 (https://gnomad.broadinstitute.org; 9), jMorp (10), and UKBB, the latter available via https://pheweb.org/UKB-TOPMed (7).
